# 5-Nitro-2,3-bis­(thio­phen-2-yl)quinoxaline

**DOI:** 10.1107/S2414314620001960

**Published:** 2020-02-14

**Authors:** Jorge F. de Freitas, Shayne Brown, James S. Oberndorfer, Guy Crundwell

**Affiliations:** a Department of Chemistry & Biochemistry, Central Connecticut State University, 1619 Stanley Street, New Britain, CT 06053, USA; Institute of Biotechnology CAS, Czech Republic

**Keywords:** crystal structure, quinoxaline, thio­phene

## Abstract

The title compound, C_16_H_9_N_3_O_2_S_2_, was synthesized *via* a condensation reaction in refluxing acetic acid. The dihedral angles between the mean plane of the quinoxaline unit and the thienyl rings are 35.16 (5)° and 24.94 (3)°.

## Abstract 2

The structure of a nitro-bis­(thio­phen-2-yl)quinoxaline has been determined at 298 K.

## Graphical abstract

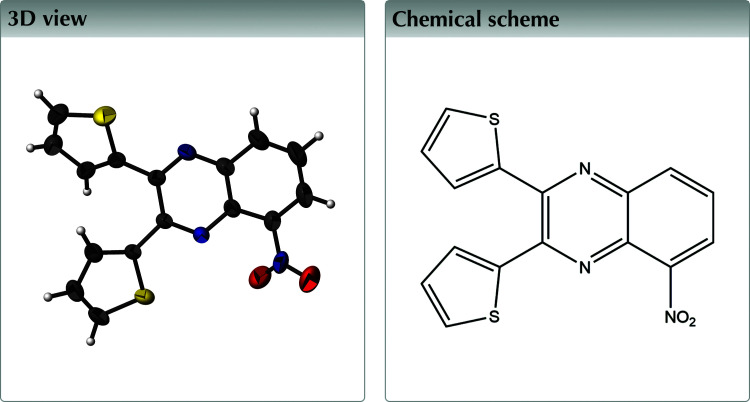

## Structure description

**Figure 1:**
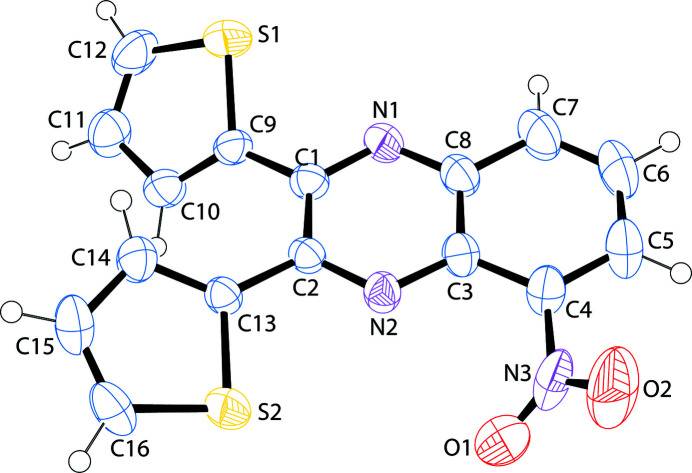
A view of 5-nitro-2,3-bis­(thio­phen-2-yl)quinoxaline (Farrugia, 2012 ▸). Displacement ellipsoids are drawn at the 50% probability level.

**Figure 2:**
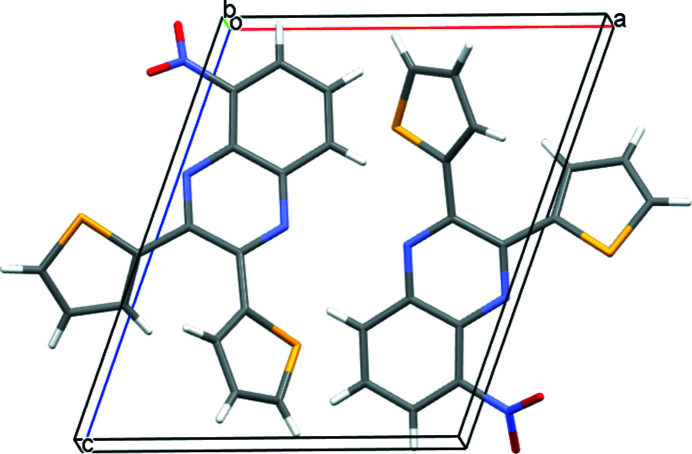
A view of the unit cell of 5-nitro-2,3-bis­(thio­phen-2-yl)quinoxaline along (010).

5-Nitro-2,3-bis­(thio­phen-2-yl)quinoxaline crystallizes in space group *P*2_1_. All bond lengths and angles are within expected values. The nitro group makes a angle of 43.07 (6)° with respect to the mean plane of the quinoxaline unit. This angle is comparable to the angles of 44.96 and 50.93° observed for the two mol­ecules in the asymmetric unit in the published crystal structure of 5-nitro-2,3-bis­(2-pyrid­yl)quinoxaline (Du & Zhao, 2003 ▸) and with the 44.12° determined in a corresponding silver complex with the pyridyl ligand (Liu & Du, 2002 ▸). The thienyl rings make angles of 35.16 (5) and 24.94 (3)°, for rings with S1 and S2 respectively, with the mean plane of the quinoxaline unit. Both the heterocyclic thienyl ring sulfur atoms reside in close proximity to the quinoxaline N atoms. When describing the structure of 5-nitro-2,3-bis­(2-pyrid­yl)quinoxaline, Du & Zhao (2003 ▸) labeled this orientation of the heterocyclic ring to the quinoxaline unit as a *trans*–*trans* arrangement. There are no inter­molecular inter­actions of consequence. An *ORTEP* view is shown in Fig. 1 ▸ and a view of the unit cell along (010) is shown in Fig. 2 ▸.

## Synthesis and crystallization

2-Thio­phene­carboxaldehyde was condensed to 2,2′-thenoin (Crundwell *et al.*, 2002 ▸) followed by oxidation to 2,2′-thenil (Crundwell *et al.*, 2003 ▸). The nitro­phenyl­enedi­amines were used as purchased from Sigma–Aldrich.

In a 100 ml round-bottom flask, 2.22 g of 2,2′-thenil (10.0 mmol) and 1.52 g of 3-nitro-1,2-phenyl­enedi­amine were added to 50 ml of concentrated acetic acid. The solution was refluxed with stirring for 18 h. The solution was cooled to room temperature and neutralized with 6 *M* NaOH. The solution was again cooled then filtered. The resulting solid was filtered and washed with cold water then dried. The yield of the yellow product was 2.80 g (83%), m.p. 445 K. Crystals were obtained by recrystallization from ethanol solution. ^1^H NMR (CDCl_3_, 300 MHz): δ = 7.17 (*m*, 2H), 7.42 (*dd*, 1H), 7.49 (*dd*, 1H), 7.80 (*m*, 2H), 7.97 (*t*, 1H), 8.30 (*m*, 2H); ^13^C NMR (CDCl_3_, 300 MHz): δ = 124.4, 127.7, 127.8, 128.8, 130.2, 130.3, 130.5, 131.0, 132.1, 132.8, 140.1, 140.5, 141.0, 147.0, 148.0, 148.1.

## Refinement

Crystal data, data collection and structure refinement details are summarized in Table 1 ▸.

## Supplementary Material

Crystal structure: contains datablock(s) 10gc15. DOI: 10.1107/S2414314620001960/ff4033sup1.cif

CCDC reference: 1983314

Additional supporting information:  crystallographic information; 3D view; checkCIF report

## Figures and Tables

**Table 1:** Experimental details

Crystal data	
Chemical formula	C_16_H_9_N_3_O_2_S_2_
*M*_r_	339.38
Crystal system, space group	Monoclinic, *P*2_1_
Temperature (K)	293
*a*, *b*, *c* (Å)	9.6598 (4), 7.4249 (3), 11.2457 (6)
β (°)	109.745 (5)
*V* (Å^3^)	759.15 (6)
*Z*	2
Radiation type	Mo *K*α
μ (mm^−1^)	0.36
Crystal size (mm)	0.42 × 0.34 × 0.21

Data collection	
Diffractometer	Oxford Diffraction Xcalibur, Sapphire3
Absorption correction	Multi-scan (*CrysAlis PRO*; Oxford Diffraction, 2009 ▸)
*T*_min_, *T*_max_	0.865, 1.000
No. of measured, independent and observed [*I* > 2σ(*I*)] reflections	12526, 6073, 3455
*R*_int_	0.021
(sin θ/λ)_max_ (Å^−1^)	0.802

Refinement	
*R*[*F* ^2^ > 2σ(*F* ^2^)], *wR*(*F* ^2^), *S*	0.038, 0.076, 0.81
No. of reflections	6073
No. of parameters	208
No. of restraints	1
H-atom treatment	H-atom parameters constrained
Δρ_max_, Δρ_min_ (e Å^−3^)	0.43, −0.15
Absolute structure	Flack (1983 ▸)
Absolute structure parameter	0.02 (4)

Computer programs: *CrysAlis CCD* and *CrysAlis RED* (Oxford Diffraction, 2009 ▸), *SHELXS2014* (Sheldrick, 2008 ▸), *SHELXL2014* (Sheldrick, 2008 ▸), *ORTEP-3 for Windows* (Farrugia, 2012 ▸) and *OLEX2* (Bourhis *et al.*, 2015 ▸).

## full crystallographic data

### Crystal data

**Table:**

C_16_H_9_N_3_O_2_S_2_	*D*_x_ = 1.485 Mg m^−^^3^
*M**_r_* = 339.38	Melting point: 445 K
Monoclinic, *P*2_1_	Mo *K*α radiation, λ = 0.71073 Å
*a* = 9.6598 (4) Å	Cell parameters from 4761 reflections
*b* = 7.4249 (3) Å	θ = 4.2–34.7°
*c* = 11.2457 (6) Å	µ = 0.36 mm^−^^1^
β = 109.745 (5)°	*T* = 293 K
*V* = 759.15 (6) Å^3^	Block, yellow
*Z* = 2	0.42 × 0.34 × 0.21 mm
*F*(000) = 348	

### Data collection

**Table:**

Oxford Diffraction Xcalibur, Sapphire3 diffractometer	6073 independent reflections
Radiation source: Enhance (Mo) X-ray Source	3455 reflections with *I* > 2σ(*I*)
Graphite monochromator	*R*_int_ = 0.021
Detector resolution: 16.1790 pixels mm^-1^	θ_max_ = 34.7°, θ_min_ = 4.2°
ω scans	*h* = −14→15
Absorption correction: multi-scan (CrysAlis PRO; Oxford Diffraction, 2009)	*k* = −11→11
*T*_min_ = 0.865, *T*_max_ = 1.000	*l* = −17→17
12526 measured reflections	

### Refinement

**Table:**

Refinement on *F*^2^	Secondary atom site location: difference Fourier map
Least-squares matrix: full	Hydrogen site location: inferred from neighbouring sites
*R*[*F*^2^ > 2σ(*F*^2^)] = 0.038	H-atom parameters constrained
*wR*(*F*^2^) = 0.076	*w* = 1/[σ^2^(*F*_o_^2^) + (0.0401*P*)^2^] where *P* = (*F*_o_^2^ + 2*F*_c_^2^)/3
*S* = 0.81	(Δ/σ)_max_ < 0.001
6073 reflections	Δρ_max_ = 0.43 e Å^−^^3^
208 parameters	Δρ_min_ = −0.15 e Å^−^^3^
1 restraint	Absolute structure: Flack (1983)
Primary atom site location: iterative	Absolute structure parameter: 0.02 (4)

### Special details

**Table:**

Geometry. All esds (except the esd in the dihedral angle between two l.s. planes) are estimated using the full covariance matrix. The cell esds are taken into account individually in the estimation of esds in distances, angles and torsion angles; correlations between esds in cell parameters are only used when they are defined by crystal symmetry. An approximate (isotropic) treatment of cell esds is used for estimating esds involving l.s. planes.
Refinement. Refinement of F^2^ against ALL reflections. The weighted R-factor wR and goodness of fit S are based on F^2^, conventional R-factors R are based on F, with F set to zero for negative F^2^. The threshold expression of F^2^ > 2sigma(F^2^) is used only for calculating R-factors(gt) etc. and is not relevant to the choice of reflections for refinement. R-factors based on F^2^ are statistically about twice as large as those based on F, and R- factors based on ALL data will be even larger. H atoms were included in calculated positions with C-H distances of 0.93 Å and were included in the refinement in riding motion approximation with U_iso_ = 1.2 of the carrier atom.

### Fractional atomic coordinates and isotropic or equivalent isotropic displacement parameters (Å^2^)

**Table:**

	*x*	*y*	*z*	*U*_iso_*/*U*_eq_	
S1	0.47899 (5)	0.46766 (7)	0.76512 (4)	0.06021 (14)	
S2	−0.19846 (4)	0.56574 (5)	0.45980 (4)	0.04714 (10)	
O1	−0.14872 (15)	0.6115 (2)	0.11787 (12)	0.0794 (4)	
O2	−0.12755 (18)	0.3929 (3)	0.00033 (12)	0.0978 (6)	
N1	0.33895 (13)	0.50828 (18)	0.48400 (11)	0.0434 (3)	
N2	0.03464 (12)	0.50012 (16)	0.35820 (10)	0.0368 (3)	
N3	−0.07726 (18)	0.4977 (3)	0.08793 (12)	0.0608 (4)	
C1	0.24643 (14)	0.52968 (18)	0.54672 (12)	0.0358 (3)	
C2	0.08928 (14)	0.51352 (17)	0.48202 (12)	0.0324 (3)	
C3	0.13010 (16)	0.4937 (2)	0.29286 (13)	0.0390 (3)	
C4	0.08067 (17)	0.4805 (2)	0.16013 (13)	0.0473 (4)	
C5	0.1750 (2)	0.4561 (3)	0.09426 (17)	0.0645 (5)	
H5	0.1394	0.4464	0.0067	0.077*	
C6	0.3257 (2)	0.4459 (3)	0.16079 (18)	0.0727 (6)	
H6	0.3903	0.4287	0.1164	0.087*	
C7	0.3801 (2)	0.4603 (3)	0.28792 (17)	0.0647 (5)	
H7	0.4811	0.4537	0.3299	0.078*	
C8	0.28385 (16)	0.4853 (2)	0.35731 (14)	0.0435 (3)	
C9	0.31323 (14)	0.5671 (2)	0.68209 (12)	0.0378 (3)	
C10	0.26900 (17)	0.6793 (2)	0.75847 (14)	0.0447 (4)	
H10	0.1831	0.7472	0.7308	0.054*	
C11	0.36745 (19)	0.6812 (2)	0.88351 (15)	0.0554 (4)	
H11	0.3529	0.7491	0.9478	0.066*	
C12	0.48426 (19)	0.5748 (3)	0.90005 (15)	0.0630 (5)	
H12	0.5599	0.5610	0.9769	0.076*	
C13	−0.02054 (14)	0.50573 (18)	0.54585 (12)	0.0332 (3)	
C14	−0.01082 (17)	0.4470 (2)	0.66529 (14)	0.0452 (4)	
H14	0.0756	0.4075	0.7262	0.054*	
C15	−0.1486 (2)	0.4545 (3)	0.68296 (16)	0.0554 (4)	
H15	−0.1625	0.4191	0.7574	0.066*	
C16	−0.25747 (19)	0.5175 (2)	0.58246 (17)	0.0552 (5)	
H16	−0.3539	0.5329	0.5802	0.066*	

### Atomic displacement parameters (Å^2^)

**Table:**

	*U*^11^	*U*^22^	*U*^33^	*U*^12^	*U*^13^	*U*^23^
S1	0.0398 (2)	0.0812 (3)	0.0524 (2)	0.0186 (2)	0.00605 (16)	0.0062 (2)
S2	0.03174 (17)	0.0601 (2)	0.0503 (2)	0.00166 (19)	0.01476 (14)	−0.0002 (2)
O1	0.0607 (8)	0.1124 (12)	0.0540 (8)	0.0145 (9)	0.0049 (6)	−0.0004 (8)
O2	0.0919 (11)	0.1455 (15)	0.0512 (8)	−0.0414 (11)	0.0180 (7)	−0.0355 (9)
N1	0.0313 (5)	0.0576 (8)	0.0441 (7)	0.0034 (5)	0.0163 (5)	0.0023 (6)
N2	0.0324 (5)	0.0459 (7)	0.0336 (6)	−0.0032 (5)	0.0130 (4)	−0.0010 (5)
N3	0.0650 (9)	0.0883 (11)	0.0270 (6)	−0.0192 (9)	0.0130 (6)	−0.0009 (7)
C1	0.0309 (6)	0.0381 (9)	0.0380 (7)	0.0006 (5)	0.0110 (5)	0.0010 (6)
C2	0.0299 (6)	0.0335 (7)	0.0359 (7)	0.0008 (5)	0.0137 (5)	0.0011 (5)
C3	0.0416 (7)	0.0408 (7)	0.0388 (7)	−0.0044 (7)	0.0193 (6)	−0.0011 (6)
C4	0.0517 (9)	0.0560 (9)	0.0362 (7)	−0.0060 (8)	0.0172 (6)	−0.0046 (7)
C5	0.0788 (13)	0.0810 (12)	0.0454 (9)	−0.0026 (12)	0.0363 (9)	−0.0057 (10)
C6	0.0749 (13)	0.0977 (15)	0.0651 (12)	0.0070 (12)	0.0494 (11)	−0.0044 (11)
C7	0.0481 (9)	0.0942 (14)	0.0624 (11)	0.0057 (10)	0.0326 (8)	−0.0038 (11)
C8	0.0393 (7)	0.0503 (8)	0.0463 (8)	0.0020 (7)	0.0214 (6)	−0.0015 (7)
C9	0.0300 (6)	0.0451 (7)	0.0361 (7)	0.0024 (7)	0.0085 (5)	0.0068 (7)
C10	0.0380 (8)	0.0513 (9)	0.0404 (8)	0.0033 (7)	0.0074 (6)	−0.0021 (7)
C11	0.0552 (10)	0.0658 (11)	0.0422 (9)	−0.0047 (9)	0.0126 (7)	−0.0075 (8)
C12	0.0515 (9)	0.0874 (13)	0.0394 (8)	0.0023 (11)	0.0014 (7)	0.0092 (10)
C13	0.0286 (6)	0.0369 (7)	0.0355 (7)	−0.0008 (5)	0.0125 (5)	−0.0013 (6)
C14	0.0446 (8)	0.0523 (9)	0.0424 (8)	−0.0013 (8)	0.0195 (6)	0.0057 (7)
C15	0.0598 (10)	0.0686 (11)	0.0483 (9)	−0.0147 (10)	0.0321 (8)	−0.0071 (9)
C16	0.0420 (8)	0.0686 (12)	0.0664 (11)	−0.0068 (8)	0.0331 (8)	−0.0123 (9)

### Geometric parameters (Å, º)

**Table:**

S1—C9	1.7234 (14)	C5—C6	1.396 (3)
S1—C12	1.6986 (19)	C6—H6	0.9300
S2—C13	1.7219 (14)	C6—C7	1.351 (2)
S2—C16	1.6992 (17)	C7—H7	0.9300
O1—N3	1.209 (2)	C7—C8	1.414 (2)
O2—N3	1.220 (2)	C9—C10	1.365 (2)
N1—C1	1.3218 (17)	C10—H10	0.9300
N1—C8	1.3528 (19)	C10—C11	1.407 (2)
N2—C2	1.3155 (17)	C11—H11	0.9300
N2—C3	1.3602 (17)	C11—C12	1.338 (2)
N3—C4	1.472 (2)	C12—H12	0.9300
C1—C2	1.4498 (18)	C13—C14	1.3849 (19)
C1—C9	1.4653 (19)	C14—H14	0.9300
C2—C13	1.4693 (18)	C14—C15	1.411 (2)
C3—C4	1.409 (2)	C15—H15	0.9300
C3—C8	1.417 (2)	C15—C16	1.341 (3)
C4—C5	1.367 (2)	C16—H16	0.9300
C5—H5	0.9300		
			
C12—S1—C9	91.46 (8)	N1—C8—C3	120.30 (12)
C16—S2—C13	91.95 (8)	N1—C8—C7	119.98 (14)
C1—N1—C8	118.73 (12)	C3—C8—C7	119.66 (14)
C2—N2—C3	118.14 (12)	C1—C9—S1	118.96 (11)
O1—N3—O2	124.17 (17)	C10—C9—S1	110.59 (10)
O1—N3—C4	119.36 (15)	C10—C9—C1	130.37 (13)
O2—N3—C4	116.47 (19)	C9—C10—H10	123.7
N1—C1—C2	120.28 (12)	C9—C10—C11	112.66 (14)
N1—C1—C9	115.93 (12)	C11—C10—H10	123.7
C2—C1—C9	123.78 (12)	C10—C11—H11	123.6
N2—C2—C1	121.01 (12)	C12—C11—C10	112.76 (15)
N2—C2—C13	114.61 (11)	C12—C11—H11	123.6
C1—C2—C13	124.37 (12)	S1—C12—H12	123.7
N2—C3—C4	121.73 (13)	C11—C12—S1	112.51 (12)
N2—C3—C8	120.66 (13)	C11—C12—H12	123.7
C8—C3—C4	117.41 (12)	C2—C13—S2	117.62 (10)
C3—C4—N3	119.71 (13)	C14—C13—S2	110.76 (10)
C5—C4—N3	117.98 (14)	C14—C13—C2	131.50 (13)
C5—C4—C3	122.29 (15)	C13—C14—H14	124.3
C4—C5—H5	120.6	C13—C14—C15	111.45 (14)
C4—C5—C6	118.83 (16)	C15—C14—H14	124.3
C6—C5—H5	120.6	C14—C15—H15	123.1
C5—C6—H6	119.1	C16—C15—C14	113.81 (14)
C7—C6—C5	121.71 (15)	C16—C15—H15	123.1
C7—C6—H6	119.1	S2—C16—H16	124.0
C6—C7—H7	120.0	C15—C16—S2	112.01 (12)
C6—C7—C8	120.09 (17)	C15—C16—H16	124.0
C8—C7—H7	120.0		
